# Controlling epidemics with transmissible vaccines

**DOI:** 10.1371/journal.pone.0196978

**Published:** 2018-05-10

**Authors:** Scott L. Nuismer, Ryan May, Andrew Basinski, Christopher H. Remien

**Affiliations:** 1 Department of Biological Sciences, University of Idaho, Moscow, ID, United States of America; 2 Department of Mathematics, University of Idaho, Moscow, ID, United States of America; University of Waterloo, CANADA

## Abstract

As the density of human and domestic animal populations increases, the threat of localized epidemics and global pandemics grows. Although effective vaccines have been developed for a number of threatening pathogens, manufacturing and disseminating vaccines in the face of a rapidly spreading epidemic or pandemic remains a formidable challenge. One potentially powerful solution to this problem is the use of transmissible vaccines. Transmissible vaccines are capable of spreading from one individual to another and are currently being developed for a range of infectious diseases. Here we develop and analyze mathematical models that allow us to quantify the benefits of vaccine transmission in the face of an imminent or ongoing epidemic. Our results demonstrate that even a small amount of vaccine transmission can greatly increase the rate at which a naïve host population can be protected against an anticipated epidemic and substantially reduce the size of unanticipated epidemics if vaccination is initiated shortly after pathogen detection. In addition, our results identify key biological properties and implementation practices that maximize the impact of vaccine transmission on infectious disease.

## Introduction

Outbreaks of infectious disease are common, and appear to have increased in frequency within the human population over the past three decades[1]. Notable recent examples include the 2014 epidemic of Ebola in West Africa, the SARS epidemic of 2003, and the 2009 Influenza pandemic. Although effective vaccines now exist–or are being developed–for a number of diseases, manufacturing, distributing, and administering a sufficient supply of vaccine presents a formidable challenge, particularly in developing countries [2–7]. One promising approach for overcoming some of the limitations of conventional vaccines is the development of transmissible vaccines capable of spreading from one individual to the next autonomously [8–10].

Transmissible vaccines have been explored and developed for wildlife (e.g., to protect rabbits against myxoma[9]), and, more recently, attention has turned to using them as a tool for eliminating human pathogens (e.g., Hantavirus and Ebola) from their animal reservoirs [8]. Although transmissible vaccines can be developed through attenuation, recent efforts have relied on recombinant vector technology that allows the antigenic genes of a target pathogen to be inserted into the genome of a transmissible, but relatively innocuous, vector organism [11]. Recent theoretical work has demonstrated that transmissible vaccines, even those which are constrained to transmit only weakly, can facilitate the eradication of endemic infectious diseases [10]. To better understand whether such weakly transmissible vaccines can have an equivalently large impact when used to prevent or minimize an impending or ongoing epidemic, we developed and analyzed simple compartmental models that coupled direct vaccination with vaccine transmission.

## Model and analyses

We began our analysis by modifying a standard model of direct vaccination to allow for vaccine transmission (S1 Text). This model assumes the host population is homogenous, well-mixed, and of a constant large size, *N*. Individuals are vaccinated directly at a steady per-capita rate, *σ*, but individuals “infected” with the vaccine are capable of transmitting the vaccine to susceptible individuals at a fixed rate, *β*_*v*_. Vaccine infected individuals are assumed to recover at a rate, *γ*_*v*_, and move into a resistant class that is immune to both the vaccine and the target pathogen. Thus, we study the best-case scenario where the vaccine leads to perfect, lifelong pathogen immunity. Although we do not specify the type of transmissible vaccine (i.e., attenuated vs. recombinant vector), our model applies to both as long as natural immunity to the vector is absent from the host population and reversion does not occur[12]. We focus our analyses on weakly transmissible vaccines with a basic reproductive number less than one (*R*_0,*v*_ < 1) because they minimize opportunities for evolution[13] and stutter to extinction when direct vaccination is ceased.

**Fig 1:**
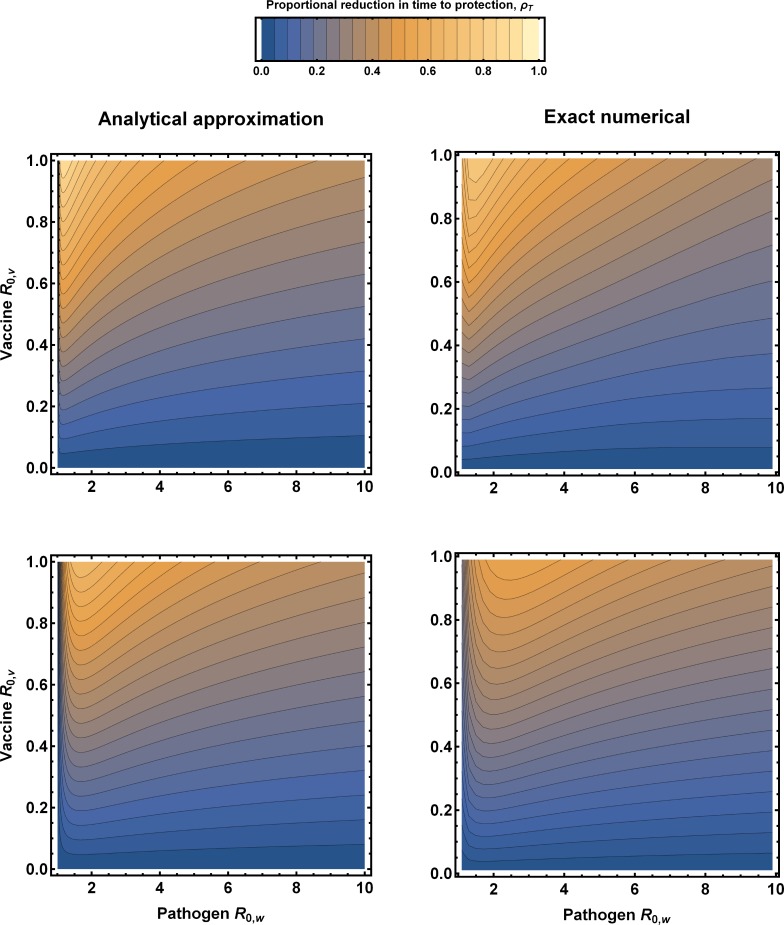
The proportional reduction in time required to vaccinate a naïve host population sufficiently to protect it from an epidemic by an infectious disease with *R*_0,*w*_ for different levels of vaccine transmission *R*_0,*v*_. The left hand column shows predictions made using the approximation (1); the right hand column shows exact numerical values. The rate of direct vaccination increases from the first row (*σ* = 0.001) to the second row (*σ* = 0.01). Individuals were assumed to recover from vaccine infection and become immune at a rate equal to *γ*_*v*_ = 0.1.

To understand how vaccine transmission influences the time required to protect a host population against an impending epidemic or pandemic (e.g., Influenza, Ebola, SARS), we developed an approximation that capitalized on our assumption of weak vaccine transmission (S1 Text). This assumption allowed us to use perturbation methods to approximate the amount of time required to fully protect a naïve host population against a pathogen with a basic reproductive number equal to *R*_0,*w*_. By comparing this quantity to the time required to protect a naïve population using a traditional, non-transmissible vaccine delivered at random, we can predict the proportion of time saved by vaccine transmission:

$$\rho_{T}\approx \frac{R_{0,v}}{R_{0,w}}\left(\frac{R_{0,w}(\sigma -\gamma_{v})+\gamma_{v}-\sigma {\left(\frac{1}{R_{0,w}}\right)}^{\frac{\gamma_{v}}{\sigma }-1}}{Log[\frac{1}{R_{0,w}}](\gamma_{v}-\sigma )}\right)$$ (1)

where *R*_0,*v*_ is the basic reproductive number of the vaccine, *γ*_*v*_ is the rate at which individuals infected with the vaccine recover and become immune, and *σ* is the rate at which the vaccine is administered to susceptible individuals. Eq (1) reveals two important results. First, even weakly transmissible vaccines can substantially reduce the time required to protect a population, reducing the time to prophylaxis by more than 50% for pathogens with modest *R*_0,*w*_ (Fig 1). Second, the proportional reduction in time required for prophylaxis is relatively insensitive to the rate of direct vaccination, becoming negligible only when rates of direct vaccination are very high and pathogen *R*_0,*w*_ very low (Fig 1; compare first and second rows). Comparison with exact numerical calculations demonstrates that the analytical approximation performs quite well, accurately capturing the qualitative relationships among parameters (Fig 1; compare left and right columns).

**Fig 2:**
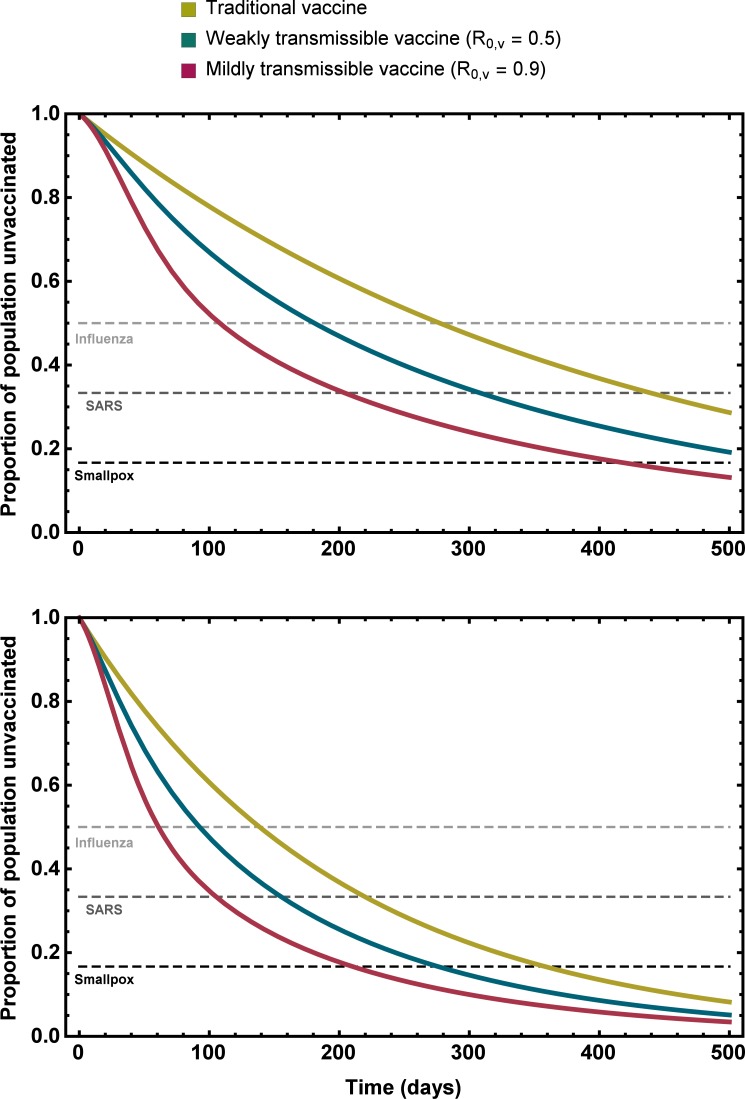
The proportion of the population remaining unvaccinated as a function of time for a traditional non-transmissible vaccine, a weakly transmissible vaccine (*R*_0,*v*_ = 0.5) and a mildly transmissible vaccine (*R*_0,*v*_ = 0.9). The dashed lines indicate the threshold proportion of the population that must be vaccinated for an epidemic/pandemic to be prevented through herd immunity assuming Influenza, SARS, and Smallpox had *R*_0_ values 2.0, 3.0, and 6.0, respectively. The rate of direct vaccination was set equal to *σ* = 0.0025 in the top panel and *σ* = 0.005 in the bottom panel.

To move beyond the qualitative insights provided by (1), and to study how significant the gains provided by a transmissible vaccine might be for real-world scenarios, we used numerical solutions of the exact equations (3) coupled with estimates for key parameters drawn from existing vaccination programs and previous epidemics/pandemics (S1 Text). These numerical solutions demonstrate that even low levels of vaccine transmission substantially reduce the time required to protect a naïve population against a set of infectious pathogens responsible for historical epidemics (Fig 2). For instance, a vaccine with *R*_0,*v*_ = 0.9 can prevent an epidemic of Influenza in ≈ 58.4% the time required by a traditional vaccine, an epidemic of SARS in ≈ 46.4% the time, and an epidemic of Smallpox in 39.0% of the time. Thus, if an epidemic is anticipated, it is not necessary for the vaccine to transmit extensively (i.e., *R*_0,*v*_ > 1) for vaccine transmission to result in a large increase in the likelihood of protecting a naïve population and preventing an epidemic through herd immunity.

In many cases it is not possible to anticipate an epidemic sufficiently far in advance for a naïve population to be sufficiently vaccinated prior to the start of an epidemic. In such cases, quantifying the benefits of vaccine transmission requires a more complex model that includes the dynamics of the infectious disease. We integrated an infectious disease into our model using a standard SIR framework and the assumption that pathogen and vaccine could not co-infect (S1 Text).

**Fig 3:**
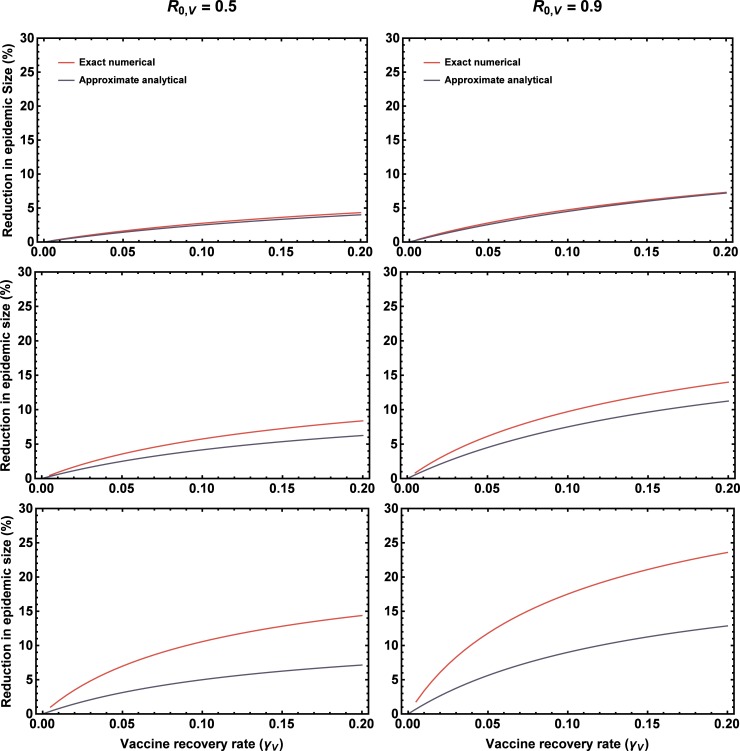
Comparison of the analytical approximation (2) and exact numerical results for the percentage reduction in epidemic size as a function of vaccine recovery rate, *γ*_*v*_ (x axis), vaccine *R*_0,*v*_ (column), and rate of direct vaccination, *σ* (rows). The rate of direct vaccination was *σ* = 0.2 in the first row, *σ* = 0.1 in the second row, and *σ* = 0.05 in the third row. In all panels *R*_0,*w*_ = 1.5 and *γ*_*w*_ = 0.1. The accuracy of the perturbation approximation falls rapidly as the rate of direct vaccination decreases and the size of pathogen outbreaks increases.

**Fig 4:**
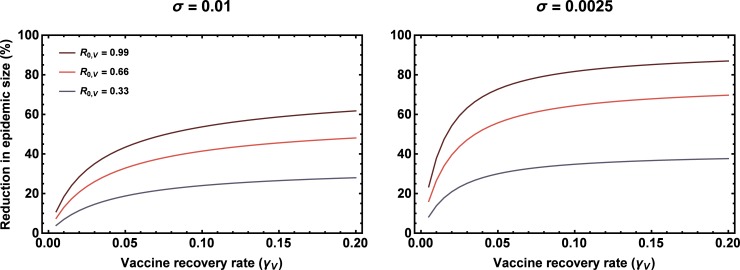
The exact percentage reduction in epidemic size as a function of vaccine recovery rate, *γ*_*v*_ (x axis), vaccine *R*_0,*v*_ (lines), and rate of direct vaccination, *σ* (column). Results calculated using numerical solutions with *R*_0,*w*_ = 1.5 and *γ*_*w*_ = 0.1 in both panels.

By assuming that both vaccine and pathogen transmitted only weakly, and that vaccination was initiated as soon as pathogen infection began, we were able to develop a perturbation solution for the final size of a small outbreak (Supporting Information: Predicting reduction in outbreak size). This solution allowed us to develop an approximation for the proportional reduction in outbreak size as a function of vaccine transmission:

$$\rho_{E}\approx \frac{\sigma \gamma_{v}\gamma_{w}R_{0,v}R_{0,w}}{\left(\sigma +\gamma_{w})(2\sigma +\gamma_{w})(\sigma +\gamma_{v}+\gamma_{w}\right)}$$ (2)

where *γ*_*w*_ is the rate at which individuals infected with the pathogen recover and become immune. This result reveals several important points. First, as expected based on simple intuition, the reduction in epidemic size increases as a function of vaccine *R*_0,*v*_ (Fig 3; compare left and right columns). Second, the relative benefits of vaccine transmission increase as the rate of direct vaccination falls (Fig 3; compare across rows). This occurs because as the rate of direct vaccination becomes large, a traditional vaccine reduces the epidemic to a small outbreak, leaving little scope for vaccine transmission to improve the situation. Third, all else being equal, as the recovery rate of the vaccine increases, so too does the proportional reduction in outbreak size (Fig 3; x axes). This latter observation has interesting implications for selecting vectors for recombinant vaccines, suggesting that transmissible vaccines designed with high rates of transmission and recovery (“fast” vaccines) will be more effective than those with an equivalent *R*_0,*v*_ but low rates of transmission and recovery. Although the analytical approximation (2) yields valuable qualitative insights, comparison with exact numerical solutions demonstrates that the approximation breaks down as the rate of direct vaccination decreases (Fig 3; compare red and blue lines). The reason the approximation fails as the rate of direct vaccination falls is because low levels of direct vaccination allow epidemics to become large and dominated by higher order terms not included in our perturbation analysis. In such cases, the solutions gleaned from our analytical approximation continue to hold qualitatively, but the reduction in epidemic size attributable to vaccine transmission becomes much larger than predicted (Fig 4). Thus, although qualitatively insightful, our approximation yields quantitatively accurate solutions only for very small outbreaks that are effectively managed by direct vaccination alone.

**Fig 5:**
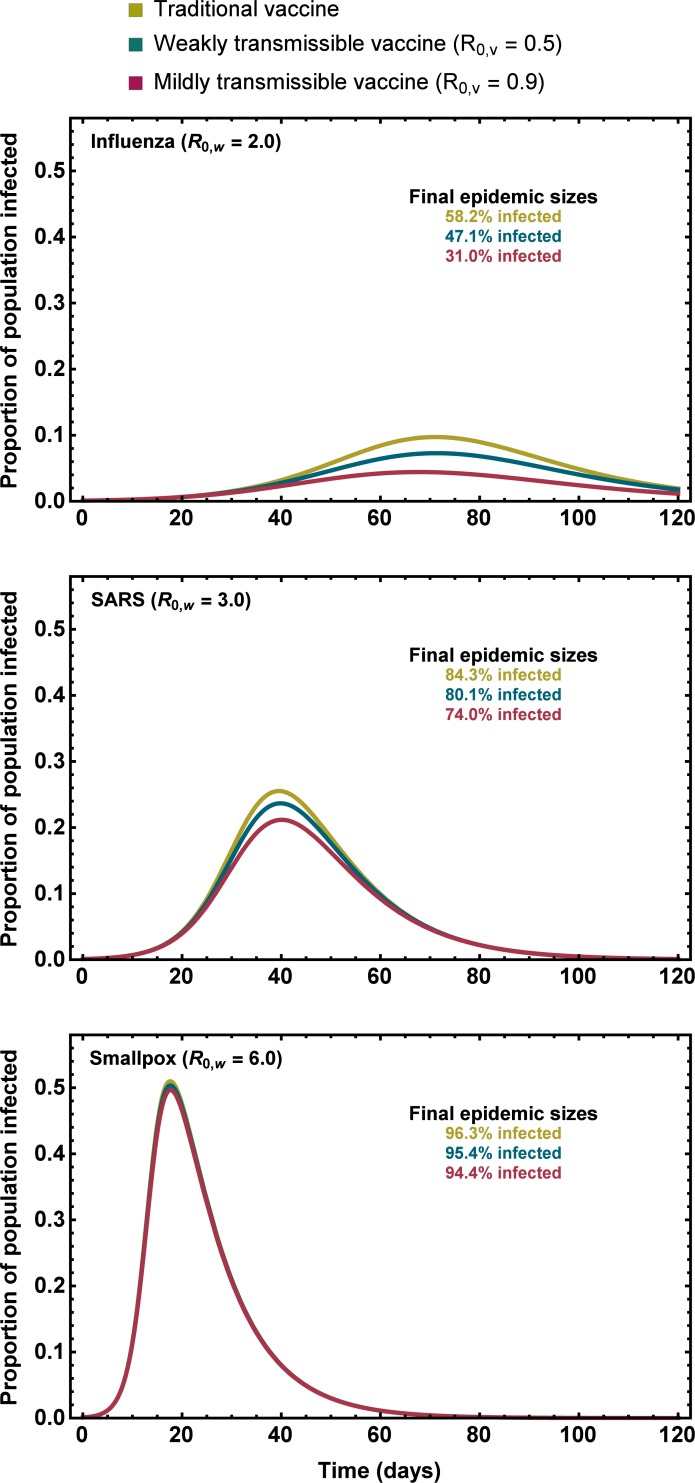
The time course of epidemics for scenarios where vaccination relies on a traditional vaccine, a transmissible vaccine with *R*_0,*v*_ = 0.5 and a transmissible vaccine with *R*_0,*v*_ = 0.9. Lines show the proportion of the host population infected at a particular time, and each panel shows a pathogen with different *R*_0,*w*_. The final size of each epidemic (% of individuals infected over the entire epidemic) is shown as inset text in each panel. The rate of direct vaccination was *σ* = 0.0025 and vaccination began at *t* = 0.

To better tie our results to pathogens with epidemic/pandemic potential, we employed numerical investigations of (3) using parameters estimated from historical epidemics/pandemics (S1 Text). The results of these numerical studies demonstrated that the qualitative conclusions of our analytical approximation are generally robust, but that the benefits of weak vaccine transmission are reduced for pathogens with relatively high *R*_0,*w*_ (Fig 5). For instance, assuming direct vaccination at rate *σ* = 0.0025, our numerical calculations demonstrate that a weakly transmissible vaccine with *R*_0,*v*_ = 0.9 can reduce the total size of an epidemic caused by a pathogen with an *R*_0,*w*_ = 2.0 (e.g., Influenza) from 58.2% to 31.0%, an epidemic caused by a pathogen with an *R*_0,*w*_ = 3.0 (e.g., SARS) from 84.3% to 74.0%, and an epidemic caused by a pathogen with an *R*_0,*w*_ = 6.0 (e.g., Smallpox) from 96.3% to 94.4%. Although these predicted reductions in epidemic size rest on a range of assumptions (e.g., no existing immunity, no heterogeneity, no alternative interventions, etc.) they suggest that even a small amount of vaccine transmission can make a large difference in the number of individuals infected by pathogens with low or modest *R*_0,*w*_ over the course of the epidemic.

**Fig 6:**
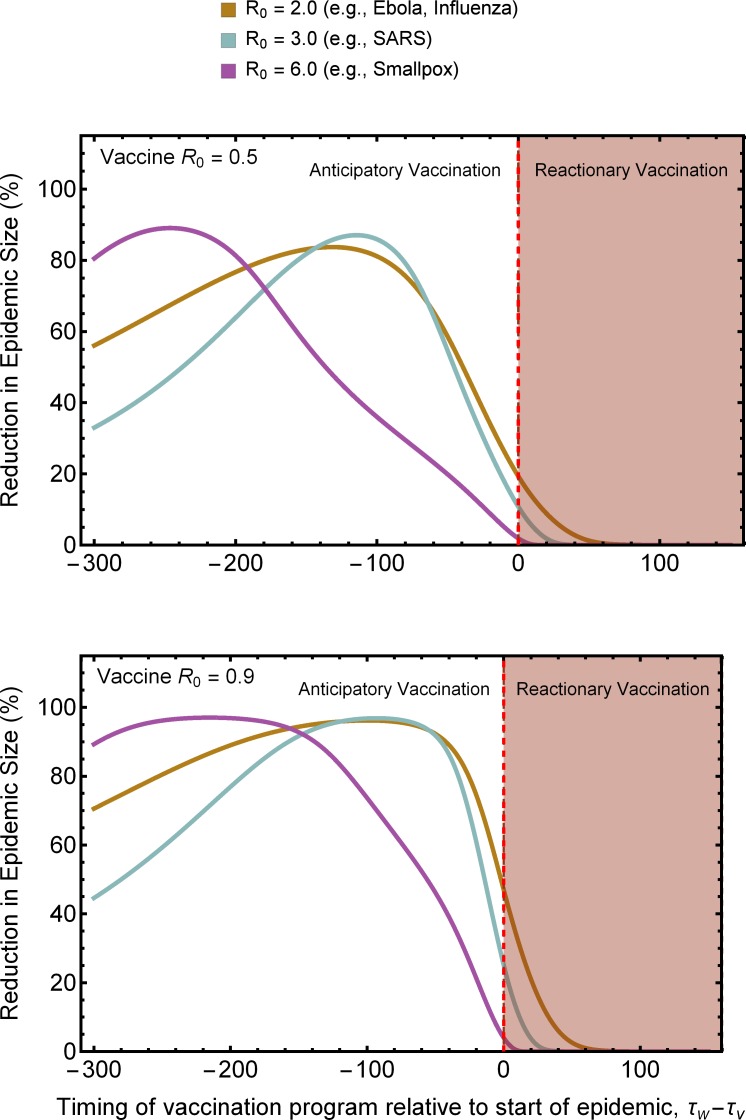
Percentage reduction in epidemic size as a function of the difference between the time at which vaccination is initiated *τ*_*V*_ and the time at which an epidemic begins *τ*_*W*_ for three different value of pathogen *R*_0,*w*_ and two different values of vaccine *R*_0,*v*_. The rate of direct vaccination was *σ* = 0.0025.

The results we have derived thus far demonstrate that transmissible vaccines can have a significant impact when used prophylactically or at the immediate outset of an epidemic. These scenarios are, of course, highly idealized, and we may more commonly be confronted with scenarios where a pathogen has already spread substantially before a vaccination program can be initiated. We generalized our results to such situations using numerical solutions of the system of ODEs (3). Specifically, we calculated the proportional reduction in epidemic size produced by a transmissible vaccine introduced at a time *τ*_*v*_ when confronted with an epidemic initiated at a time *τ*_*w*_. The results of these numerical analyses demonstrate that the benefits of vaccine transmission are very sensitive to timing, with benefits maximized when a vaccination program can be initiated prior to the start of an epidemic, but not sufficiently far in advance for a standard vaccine to be effective (Fig 6). Not surprisingly, the greater the *R*_0,*w*_ of the pathogen, the earlier the epidemic must be detected, and a vaccination program initiated, for the benefits of vaccine transmission to be fully realized. Even when the benefits of vaccine transmission are not fully realized (i.e., when an epidemic is underway before vaccination can begin) a transmissible vaccine can still substantially reduce the size of an epidemic caused by a pathogen with modest *R*_0,*w*_ (Fig 6).

**Fig 7:**
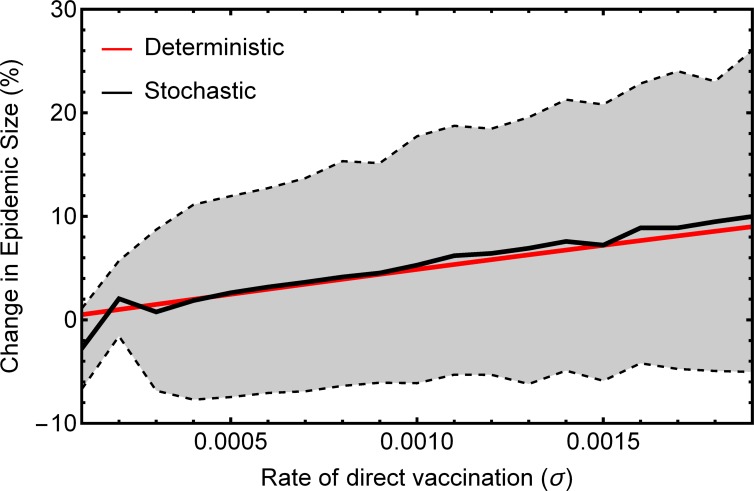
Percentage change in epidemic size as a function of the rate of direct vaccination calculated from numerical solutions to (3) and the average of 1000 stochastic simulations for each rate of direct vaccination. Dashed lines and shaded area denote the lower 5% and upper 95% of simulated values. Parameter values were: *β*_*w*_ = 0.0002, *β*_*v*_ = 0.00009, *γ*_*w*_ = 0.1, *γ*_*v*_ = 0.1, with initial population sizes S = 1000 and W = 20.

To this point we have assumed that both pathogen and vaccine are sufficiently abundant for stochastic impacts on their dynamics to be ignored. In cases where direct vaccination rates are relatively small, however, demographic stochasticity may become appreciable, potentially reducing the efficacy of vaccine transmission. In order to explore this scenario, we developed stochastic simulations (S1 Code) using the Gillespie Algorithm [14]. We used these simulations to compare our deterministic predictions for the reduction in epidemic size to that realized across 500 replicate simulations for cases where the rate of direct vaccination was small (0.0001 ≤ *σ* ≤ 0.002). Differences between deterministic predictions and the mean of stochastic simulations were small for all but the smallest of direct vaccination rates (i.e., *σ* ≤ 0.0002), suggesting that our predictions are robust even when a rare transmissible vaccine is subject to stochastic extinction (Fig 7).

## Discussion

We have used simple mathematical models to demonstrate that transmissible vaccines could help curb future epidemics. By reducing the amount of vaccine that needs to be manufactured, and the time required to disseminate it within the population, vaccine transmission could help to overcome key hurdles to effective pandemic response [15, 16]. In addition to demonstrating the potential utility of transmissible vaccines, our model clarifies the conditions under which the benefits of vaccine transmission will be maximized. Specifically, transmissible vaccines will have the greatest impact (relative to a traditional vaccine) when an epidemic is anticipated, but not sufficiently far in advance for a traditional vaccine to fully protect the population. In addition, the positive impacts of a transmissible vaccine will be greatest when used against pathogens with low *R*_0_ values, and in cases where the transmissible vaccine can be designed to have dynamics that are “fast” relative to those of the pathogen.

Although our results suggest transmissible vaccines can be effective tools in preventing or minimizing epidemics, our mathematical model relies on a number of important assumptions. First, we have assumed the vaccine offers lifelong immunity with perfect protection against the infectious disease. Although unrealistic for many vaccines and known to have important epidemiological and evolutionary consequences [17–19], this assumption is unlikely to qualitatively influence our results because we measure the benefits of vaccine transmission relative to a traditional vaccine that also offers perfect lifelong immunity. Second, our model assumes a single well-mixed population, and thus ignores the potential importance of spatial and individual heterogeneity [19–21]. Finally, our models have ignored the potential for evolution, which is likely to have different impacts depending on the type of transmissible vaccine [11]. Specifically, evolution creates the potential for attenuated transmissible vaccines to revert to wild type. In addition to the obvious negative consequences of such reversion at the individual level, reversion to wild type would effectively introduce the pathogen into the target population prematurely, thus reducing the population level benefits of vaccine transmission. In contrast, evolution in recombinant vector vaccines may result in reversion to free vector, resulting in competition with the vaccine [12], and reducing the benefits of vaccine transmission.

Although our results demonstrate substantial benefits of transmissible vaccines, there are, of course, substantial challenges and risks that must yet be confronted for their effective and safe use in real populations, many of which involve unwanted evolution [11]. The most worrisome outcome of evolution in any transmissible vaccine is the spread of mutations conferring increased virulence as has been observed in the oral polio vaccine [22–24]. Although undeniably problematic in vaccines developed through traditional attenuation, vaccines developed using recombinant vector technology should not be prone to the evolution of increased virulence, as long as an innocuous vector with a long history of association with the target host is used [11]. In such cases, unwanted evolution may pose more of a logistical challenge than risk. Specifically, transmissible recombinant vector vaccines require that the antigenic insert be stably maintained in the replicating vector population in the face of inevitable selection favoring mutations to the insert free state [12]. Overcoming this engineering challenge may prove to be a more significant obstacle to the development of transmissible vaccines than the evolution of increased virulence.

Even if transmissible vaccines are never deemed acceptable for direct use in human populations, their potential impacts on conservation, agriculture, and human health remains enormous [8, 11]. Developing a better understanding of the effectiveness of transmissible vaccines will require more complex models that integrate host heterogeneity [20] and the potential for vaccine evolution [11]. Coupling these more complex models with results of experimental studies evaluating the rate and consequences of evolution across candidate vaccine designs will establish a framework for deciding when transmissible vaccines are likely to be both effective and safe.

## Supporting information

S1 Text This file includes a detailed description of the mathematical model and derivations for all key mathematical results in the main text. Also included is a description of how key parameters were estimated.
(DOCX)

S1 Code This file includes the C++ source code for the stochastic simulations. (CPP)
