# Supplementary material for: Controlling epidemics with transmissible vaccines
Source: PLoS One. 2018 May 10;13(5):e0196978. doi: 10.1371/journal.pone.0196978 (PMC5945036; doi:10.1371/journal.pone.0196978)
Supplement: S1 Text — Also included is a description of how key parameters were estimated. (DOCX) [file pone.0196978.s001.docx]

**Supporting information**

*Model Derivation*

We extend a standard differential equation-based SIR model to include vaccine transmission by adding an additional class of individuals who are “infected” with the vaccine. Susceptible individuals (S) are directly infected by vaccine at per-capita rate $\sigma$, infected by vaccine through transmission from vaccine-infected individuals (V) at rate $\beta_{v}$, and infected by pathogen-infected individuals (W) at rate $\beta_{w}$. Vaccine infected individuals and pathogen infected individuals recover at rates $\gamma_{v}$ and $\gamma_{w}$, respectively, and transition to the vaccine-recovered and pathogen-recovered classes, $R_{v}$ and $R_{w}$, respectively. We assume standard mass action transmission functional responses for both the vaccine and the pathogen. For simplicity, we do not include pathogen virulence, and assume a closed population of constant size. These assumptions lead to the following system of differential equations:

$\frac{dS}{dt}=-\sigma S-\beta_{v}SV-\beta_{w}SW$ (3a)

$\frac{dV}{dt}=\sigma S+\beta_{v}SV-\gamma_{v}V$ (3b)

$\frac{dW}{dt}=\beta_{w}SW-\gamma_{w}W$ (3c)

$\frac{dR_{v}}{dt}=\gamma_{v}V$ (3d)

$\frac{dR_{w}}{dt}=\gamma_{w}W$ (3e)

*Non-dimensionalization*

We non-dimensionalize equations (3) by scaling state variables by total population size, $N=S+V+W+R_{v}+R_{w}$, and time by $\gamma_{w}$. Letting $s=S/N$, $v=V/N$, $w=W/N$, $r_{v}=R_{v}/N$, $r_{w}=R_{w}/N$, and $=\gamma_{w}t$ yields the following system of differential equations:

$\frac{ds}{d}=-s-R_{0,v}sv-R_{0,w}sw$ (4a)

$\frac{dv}{d}=s+R_{0,v}sv-v$ (4b)

$\frac{dw}{d}=R_{0,w}sw-w$ (4c)

$\frac{dr_{v}}{d}=v$ (4d)

$\frac{dr_{w}}{d}=w$ (4e)

where $=\sigma/\gamma_{w}$, $=\gamma_{v}/\gamma_{w}$, $R_{0,v}=\beta_{v}N/\gamma_{v}$, and $R_{0,w}=\beta_{w}N/\gamma_{w}$.

*Prophylactic Vaccination*

In this section, we are interested in deriving an approximation for the time to prophylaxis against an impending disease. Here, the pathogen is not yet present in the population so $w=0$, and the governing equations (4) simplify to

$\frac{ds}{d}=-s-R_{0,v}sv$ (5a)

$\frac{dv}{d}=s+R_{0,v}sv-v$ (5b)

$\frac{dr_{v}}{d}=v$. (5c)

We assume that all individuals are initially susceptible so that $s(0)=1$ and $v\left( 0 \right)=r_{v}(0)=0$.

We will find an approximate solution to (5) by performing a perturbation expansion around low vaccine transmission $R_{0,v}$. We begin by expanding $s$, $v$, and $r_{v}$ in powers of $R_{0,v}$ so that

$s\left( ;R_{0,v} \right)=s_{0}\left( \right)+R_{0,v}s_{1}\left( \right)+ O(R_{0,v}^{2})$ (6a)

$v\left( ;R_{0,v} \right)=v_{0}\left( \right)+R_{0,v}v_{1}\left( \right)+ O(R_{0,v}^{2})$ (6b)

$r_{v}\left( ;R_{0,v} \right)=r_{v,0}\left( \right)+R_{0,v}r_{v,1}\left( \right)+ O\left( R_{0,v}^{2} \right)$. (6c)

Substituting (6) into equations (5) and setting $R_{0,v}=0$ yields the $O\left( 1 \right)$ equations:

$\frac{ds_{0}}{d}=-s_{0}$ (7a)

$\frac{dv_{0}}{d}=s_{0}-v_{0}$ (7b)

$\frac{dr_{v,0}}{d}=v_{0}$ (7c)

with initial conditions $s_{0}\left( 0 \right)=1$ and $v_{0}\left( 0 \right)=r_{v,0}(0)=0$, which have the following solution:

$s_{0}\left( \right)=e^{-}$ (8a)

$v_{0}\left( \right)=-\frac{\left( e^{-}-e^{-} \right)}{-}$ (8b)

$r_{v,0}\left( \right)=\frac{\left( 1-e^{-} \right)+(-1+e^{-})}{-}$ . (8c)

We now find solutions to the $O\left( R_{0,v} \right)$ equations by substituting the expansion (6) and the $O\left( 1 \right)$ solutions (8) into equations (5) to get the following $O\left( R_{0,v} \right)$ system of equations

$\frac{ds_{1}}{d}=-s_{1}+\frac{e^{-(+2)}\left( {-e}+e \right)}{-}$ (9a)

$\frac{dv_{1}}{d}=s_{1}+\frac{{((e}^{-2}-e^{-\left( + \right)})+(-+))}{-}v_{1}$ (9b)

$\frac{dr_{v,1}}{d}=v_{1}$ (9c)

with $s_{1}\left( 0 \right)=v_{1}\left( 0 \right)=r_{v,1}\left( 0 \right)=0$, which we solved for $s_{1}\left( \right)$, $v_{1}\left( \right)$, and $r_{v,1}\left( \right)$.

Substituting the $O\left( 1 \right)$ and $O\left( R_{0,v} \right)$ solutions into the expansion (6) and dropping higher order terms gives the following approximation for $v_{tot}\left( \right)=v\left( \right)+r_{v}\left( \right)$, the total fraction vaccinated at time ,

$v_{tot}\left( \right)\approx\frac{e^{-\left( +3 \right)}(-e^{\left( + \right)}R_{0,v}+e^{\left( +3 \right)}\left( - \right)+e^{\left( +2 \right)}\left( R_{0,v}-1 \right)\left( - \right)+e^{2}R_{0,v})}{-}.$ (10)

Equation (10) gives the relationship between the total fraction of vaccinated individuals $v_{tot}$ and time . Unfortunately, cannot be solved for explicitly in this equation to give an expression for ${}_{c}$, the critical time required to vaccinate $v_{tot}$ fraction of individuals. Instead, we seek an approximation for ${}_{c}$, again by expanding about low vaccine transmission. We wish to find the time ${}_{c}$ that solves $v_{tot}\left( {}_{c}\left( R_{0,v} \right), R_{0,v} \right)=v_{crit}$ where $v_{crit}=1-1/R_{0,w}$ is the fraction of the population that must be vaccinated to prevent invasion of a pathogen with basic reproductive number $R_{0,w}$.

To approximate ${}_{c}$, we expand about $R_{0,v}=0$ so that ${}_{c}\left( R_{0,v} \right)={}_{c}\left( 0 \right)+\frac{d{}_{c}(0)}{dR_{0,v}}R_{0,v}+ O(R_{0,v}^{2})$. Setting $R_{0,v}=0$ in equation (10) and solving for , we find that ${}_{c}\left( 0 \right)=-log(1-v_{crit})/$. To find $\frac{d{}_{c}}{dR_{0,v}}$, we use implicit differentiation. Differentiating both sides of $v_{tot}\left( {}_{c}\left( R_{0,v} \right), R_{0,v} \right)=v_{crit}$ yields $\frac{dv_{tot}}{d{}_{c}}\frac{d{}_{c}}{dR_{0,v}}+\frac{dv_{tot}}{dR_{0,v}}=0$ so that $\frac{d{}_{c}}{dR_{0,v}}=-(\frac{dv_{tot}}{dR_{0,v}})/(\frac{dv_{tot}}{d{}_{c}})$. We evaluate $\frac{dv_{tot}}{dR_{0,v}}$ and $\frac{dv_{tot}}{d{}_{c}}$ by differentiating (10) with respect to $R_{0,v}$ and ${}_{c}$, respectively, substitute our solution for ${}_{c}$ at $R_{0,v}=0$, and evaluate the resulting expressions at $R_{0,v}=0$, which yields:

$\frac{d{}_{c}(0)}{dR_{0,v}}\approx\frac{v_{crit}+\left( -1+\left( 1-v_{crit} \right)^{/} \right)}{(-+)}$. (11)

Dropping higher order terms and combining the $O\left( 1 \right)$ and $O\left( R_{0,v} \right)$ solutions yields the following approximation for the time required to vaccinate a critical fraction $v_{crit}$ of the population:

${}_{c}\approx-\frac{\log\left( 1-v_{crit} \right)}{}+ \frac{v_{crit}+\left( \left( 1-v_{crit} \right)^{/}-1 \right)}{(-)}R_{0,v}$. (12)

Substituting $v_{crit}=1-\frac{1}{R_{0,w}}$, we can write expression (12) in terms of the pathogen’s basic reproductive number to give an expression for the time required to vaccinate a population to a sufficient extent so as to prevent invasion by a pathogen with basic reproductive number $R_{0,w}$:

${}_{c}(R_{0,v})\approx-\frac{\log\left( \frac{1}{R_{0,w}} \right)}{}+ \frac{\frac{{(R}_{0,w}-1)}{R_{0,w}}+\left( -1+\left( 1/R_{0,w} \right)^{/} \right)}{(-)}R_{0,v}$. (13)

With an approximation for ${}_{c}$ now in hand, the proportion of time saved by vaccination defined as $\rho_{T}=\frac{{}_{c}(0)-{}_{c}(R_{0,v})}{{}_{c}(0)}$ is found to be

$\rho_{T}\approx\frac{R_{0,v}}{R_{0,w}}\left( \frac{R_{0,w}\left( \sigma-\gamma_{v} \right)+\gamma_{v}-\sigma\left( \frac{1}{R_{0,w}} \right)^{\frac{\gamma_{v}}{\sigma}-1}}{\mathrm{Log}\left[ \frac{1}{R_{0,w}} \right](\gamma_{v}-\sigma)} \right)$ (14)

*Predicting the reduction in outbreak size*

We use a perturbation analysis to find an approximation for how the final size of a small outbreak varies with vaccination rate and vaccine transmission. Our general approach is to develop an approximation that relies on the assumption of both small vaccine and pathogen $R_{0}$. A consequence of this assumption is that our approximation is likely to be quantitatively accurate only for very small outbreaks of infectious disease controlled by very weakly transmissible vaccines. Our hope, however, is that the qualitative insights this approximation provides will be robust for larger outbreaks and epidemics. We find an approximation to (4) by performing a perturbation expansion about small $R_{0,v}$ and low $R_{0,w}$, assuming that both $R_{0,v}$ and $R_{0,w}$ are of similar small order $R_{0}$. Letting $R_{0,v}={c_{1}R}_{0}$ and $R_{0,w}={c_{2}R}_{0}$ for $O\left( 1 \right)$ constants $c_{1}$ and $c_{2}$, we can rewrite equations (4) as

$\frac{ds}{d}=-s-{c_{1}R}_{0}sv-{c_{2}R}_{0}sw$ (15a)

$\frac{dv}{d}=s+{c_{1}R}_{0}sv-v$ (15b)

$\frac{dw}{d}={c_{2}R}_{0}sw-w$ (15c)

$\frac{dr_{v}}{d}=v$ (15d)

$\frac{dr_{w}}{d}=w$ (15e)

As we are only concerned with the total number infected, we do not need to track $r_{v}$ throughout this analysis. We expand the state variables in powers of $R_{0}$; a second-order solution is needed to capture interaction between vaccine and pathogen-infected individuals:

$s\left( ;R_{0} \right)=s_{0}\left( \right)+R_{0}s_{1}\left( \right)+R_{0}^{2}s_{2}\left( \right)+ O(R_{0}^{3})$ (16a)

$v\left( ;R_{0} \right)=v_{0}\left( \right)+R_{0}v_{1}\left( \right)+R_{0}^{2}v_{2}\left( \right)+ O(R_{0}^{3})$ (16b)

$w\left( ;R_{0} \right)=w_{0}\left( \right)+R_{0}w_{1}\left( \right)+R_{0}^{2}w_{2}\left( \right)+ O(R_{0}^{3})$ (16c)

$r_{w}\left( ;R_{0} \right)=r_{w,0}\left( \right)+R_{0}r_{w,1}\left( \right)+R_{0}^{2}r_{w,2}\left( \right)+ O(R_{0}^{3})$ (16d)

We proceed with the perturbation analysis to find and solve the $O\left( 1 \right)$, $O\left( R_{0} \right)$, and $O\left( R_{0}^{2} \right)$ equations yielding expressions for $s_{0}\left( \right)$, $v_{0}\left( \right)$, $w_{0}\left( \right)$, $r_{w,0}\left( \right)$, $s_{1}\left( \right)$, $v_{1}\left( \right)$, $w_{1}\left( \right)$, $r_{w,1}\left( \right)$ and $s_{2}\left( \right)$, $v_{2}\left( \right)$, $w_{2}\left( \right)$, $r_{w,2}\left( \right)$, respectively; these expressions are omitted for brevity.

To find an approximate expression for the fraction of the population infected by the end of the epidemic as a function of vaccine transmission, $r_{w,total}\left( R_{0,v} \right),$we substitute the expressions for $r_{w,0}\left( \right)$, $r_{w,1}\left( \right)$, and $r_{w,2}\left( \right)$ into the expansion (16d), drop $O\left( R_{0}^{3} \right)$ terms, and take the limit as tends to infinity. We define the fractional reduction in disease incidence as

$\rho_{E}=\frac{r_{w,total}\left( 0 \right)-r_{w,total}\left( R_{0,v} \right)}{r_{w,total}\left( 0 \right)}$. Assuming that initial pathogen incidence is low and most individuals are initially susceptible, we set $s\left( 0 \right)=1$, yielding the expression

$\rho_{E}\approx\frac{c_{1}c_{2}R_{0}^{2}}{(1++)(1+3+2{}^{2})}$ (19)

which in terms of our non-dimensional parameters can be written

$\rho_{E}\approx\frac{R_{0,v}R_{0,w}}{(1++)(1+3+2{}^{2})}$. (20)

Converting and to our original parameters,$\rho_{E}$ can be written as

$\rho_{E}\approx\frac{R_{0,v}R_{0,w}{}_{v}{}_{w}\sigma}{({}_{w}+\sigma)({}_{w}+{}_{v}+\sigma)({}_{w}+2\sigma)}$. (21)

All calculations were performed using Mathematica version 11.0.1.0 and code is available as supplemental information.

*Parameter values*

We tethered our results to pathogens responsible for recent epidemics and pandemics, or those of special concern for a future pandemic (e.g., smallpox), using published estimates of $R_{0}$ [1-5]. Not surprisingly, these published studies include a number of different estimates for $R_{0}$ for each pathogen, and the numerical results we report rely on selecting what seemed to be a representative value for each pathogen. We emphasize that our goal was not to generate precise numerical predictions, but rather to develop a qualitative understanding for the benefits of a transmissible vaccine when used against pathogens with varying $R_{0}$. Rates of direct vaccination, $\sigma$, were based on U.S. Centers for Disease Control estimates for vaccine distribution during the 2016-2017 Influenza season (<https://www.cdc.gov/flu/professionals/vaccination/vaccinesupply-2016.htm>). Specifically, we estimated a value of $\sigma$ by calculating that 149.5 million doses were distributed over a period of 182 days and dividing that number by the current population size of the United States. These calculations result in an estimate of $\sigma=0.0025 per day$.

*References*

1. Anderson RM, Fraser C, Ghani AC, Donnelly CA, Riley S, Ferguson NM, et al. Epidemiology, transmission dynamics and control of SARS: the 2002-2003 epidemic. Philosophical Transactions of the Royal Society of London Series B-Biological Sciences. 2004;359(1447):1091-105. doi: 10.1098/rstb.2004.1490. PubMed PMID: WOS:000222688200008.

2. Aylward B, Barboza P, Bawo L, Bertherat E, Bilivogui P, Blake I, et al. Ebola Virus Disease in West Africa - The First 9 Months of the Epidemic and Forward Projections. New England Journal of Medicine. 2014;371(16):1481-95. doi: 10.1056/NEJMoa1411100. PubMed PMID: WOS:000342994700005.

3. Ferguson NM, Cummings DAT, Fraser C, Cajka JC, Cooley PC, Burke DS. Strategies for mitigating an influenza pandemic. Nature. 2006;442(7101):448-52. doi: 10.1038/nature04795. PubMed PMID: WOS:000239278900042.

4. Gani R, Leach S. Transmission potential of smallpox in contemporary populations. Nature. 2001;414(6865):748-51. doi: 10.1038/414748a. PubMed PMID: WOS:000172676200047.

5. Zhou GF, Yan GY. Severe acute respiratory syndrome epidemic in Asia. Emerging Infect Dis. 2003;9(12):1608-10. PubMed PMID: WOS:000187247600017.
